# A nomogram for predicting early bacterial infection after liver transplantation: a retrospective study

**DOI:** 10.3389/fmed.2025.1563235

**Published:** 2025-04-10

**Authors:** Jie Yu, Jichang Jiang, Caili Fan, Jinlong Huo, Tingting Luo, Lijin Zhao

**Affiliations:** ^1^Department of General Surgery, Digestive Disease Hospital, Affiliated Hospital of Zunyi Medical University, Zunyi Guizhou, China; ^2^Department of Nursing, Affiliated Hospital of Zunyi Medical University, Zunyi, Guizhou, China; ^3^Department of Breast and Thyroid Surgery, The Third Affiliated Hospital of Zunyi Medical University (The First People’s Hospital of Zunyi), Zunyi, Guizhou, China

**Keywords:** bacterial infection, risk factors, systemic immune inflammation index, neutrophil to lymphocyte ratio, predictive model

## Abstract

**Background:**

Bacterial infection is a common complication of liver transplantation and is associated with high mortality rates. However, multifactor-based early-prediction tools are currently lacking. Therefore, this study investigated the risk factors of early bacterial infections after liver transplantation and used them to establish a nomogram.

**Methods:**

We retrospectively collected the clinical data of 232 patients who underwent liver transplantation. We excluded 15 patients aged less than 18 years, 7 patients with infection before transplantation, and 3 patients with incomplete laboratory test results based on the sample exclusion criteria, and finally included 207 liver transplant patients. The patients were divided into the bacterial infection group (75 cases) and non-infected group (132 cases) according to whether bacterial infection had occurred within 30 days after surgery. The associated risk factors were determined using stepwise regression, and a nomogram was established based on the results of the multifactorial analysis. The predictive performance of the model was compared by assessing the area under the receiver operating characteristic curve (AUC-ROC), decision curve analysis (DCA), and the calibration curve, which was validated using cross-validation and repeated sampling.

**Result:**

Preoperative systemic immune inflammation index (SII) (OR = 1.003, *p* = 0.001), duration of surgery (OR = 1.008, *p* = 0.005), duration of postoperative ventilator use (OR = 1.013, *p* = 0.025), neutrophil to lymphocyte ratio (NLR) (OR = 1.017, *p* = 0.024), ICU stay time (OR = 1.125, *p* = 0.015) were independent risk factors for early bacterial infection after liver transplantation. The nomogram was constructed based on the above factors, achieving an AUC of 0.863 (95%CI: 0.808, 0.918), which showed that the mean absolute error between the predicted risk and the actual risk of the model was 0.044. The decision curve analysis showed that it was located above both extreme curves in a range of more than the 14% threshold, which indicated that there was a good clinical benefit in this range. Internal validation using 10-fold cross validation and bootstrap replicate sampling yielded areas under the corrected ROC curves of 0.842 and 0.854, respectively. These results indicate that the developed model exhibits good predictive performance and a moderate error in training and validation.

**Conclusion:**

The nomogram constructed in this study showed good differentiation, calibration, and clinical applicability. It can effectively identify the high-risk group for bacterial infection in the early postoperative period after liver transplantation, while simultaneously helping the transplant team dynamically monitor the key indicators and optimize perioperative management.

## 1 Introduction

Liver transplantation (LT) is the most effective treatment for end-stage liver disease at this stage (1). The management of postoperative complications is challenging because of the patient’s low preoperative baseline immune function, high nutritional risk, possible combination of multiple organ failure, and postoperative immunosuppression (2). Among all solid organ transplants, liver transplant patients have the highest rate of postoperative bacterial infections at 30% ~ 70% (3, 4), especially during the first month after transplantation, which not only prolongs the duration of hospitalization and increases the financial burden of the patients but is also a major cause of early mortality (5). One study reported that LT patients with combined bacterial infections had a mortality rate of 38.9% within 30 days of surgery (6), and this high mortality rate further emphasized the important impact of early postoperative bacterial infections on the prognosis of LT patients. However, early post-transplant infections and rejection overlap at the time of onset and clinical manifestations (7, 8), making it difficult for clinicians to quickly identify the two, making clinical immune function modulation and anti-infection therapy a dilemma. Notably, the routine use of immunosuppressive agents after transplantation places the patients’ immune functions in a state of long-term suppression, leading to a significant increase in the risk of infection by various pathogenic microorganisms, especially during the early administration of medication or hormone shock therapy. Selimoğlu et al. (9) found that immunosuppressive agents increased the risk of total postoperative infections, and the risk of bacterial infections by 5.3-fold and 2.5-fold, respectively. In conclusion, based on the complexity of immune function regulation after liver transplantation, clarifying the independent risk factors for postoperative bacterial infections after LT and early and accurate prediction of bacterial infections have a positive impact on improving the prognosis of LT patients.

Currently, the standard for diagnosing infections is microbial culture; however, the culture time is long, rate of contamination is high, and positivity rate is low (10, 11). C-reactive protein (CRP), an acute-phase reactive protein, has a diagnostic value in infections. However, it is detected after the inflammatory process occurs for approximately 12 h, and its specificity and sensitivity are lower than those of procalcitonin (PCT); therefore, the application of serum CRP to diagnose bacterial infections cannot be performed quickly and accurately after the onset of the disease, thus limiting the value of CRP in diagnosing early bacterial infections, especially severe bacterial infections (12). The PCT levels are more specific for the diagnosis of bacterial infections; however, several studies have shown that PCT levels are elevated in the early postoperative period after LT with or without infection, and that its early diagnostic value is susceptible to factors including donor category, site of infection, surgical trauma (13, 14). Cousin et al. (15) concluded that PCT levels during the first postoperative week after LT are not useful for diagnosing bacterial infections without any significance. In recent years, Metagenomic Next-generation sequencing (mNGS) has been widely used, with fast detection speed, high sensitivity, and high throughput, which is much higher than those in the traditional microbial culture in terms of pathogenic bacteria detection rate and detected strains (16); however, it also has high cost, lack of judgment standards, and high false-positive rate limitations, which need to be further adapted and improved (17). Therefore, this study aimed to develop a simple, accurate, and specific tool for the early prediction of bacterial infections after LT surgery that will help clinicians implement targeted treatment plans.

A nomogram is a visualization tool based on statistical models, which usually adopts a quantitative approach to predict the probability of an individual’s certain outcome event occurring in the future and is more stable in small sample data than in machine learning models. In addition, in clinical practice, nomograms are easy to calculate and visualize, which can support immediate decision-making for doctors and patients, and are suitable for clinical bedside assessment. In this study, we evaluated and validated the model by analyzing the risk factors for postoperative bacterial infections after LT and used them to construct a risk prediction model for early postoperative bacterial infections after LT. The results of this study will be conducive to the clinical screening of high-risk patients for postoperative bacterial infection, revealing the risk factors and key aspects of post-LT bacterial infection, providing a reference basis for the targeted implementation of preventive measures, and thus effectively reducing the incidence of post-LT bacterial infections.

## 2 Materials and methods

### 2.1 Patients

We selected patients who underwent LT surgery at the Affiliated Hospital of Zunyi Medical University from November 2019 to October 2024, totaling 232 patients, as study participants. All the required data and information were extracted from the electronic medical record management system and transplantation database, which did not involve the administration of drugs or other clinical interventions to the patients and were not potentially harmful. During the study, the patients’ personal information was completely anonymized and de-identified to ensure privacy and confidentiality.

**Figure 1:**
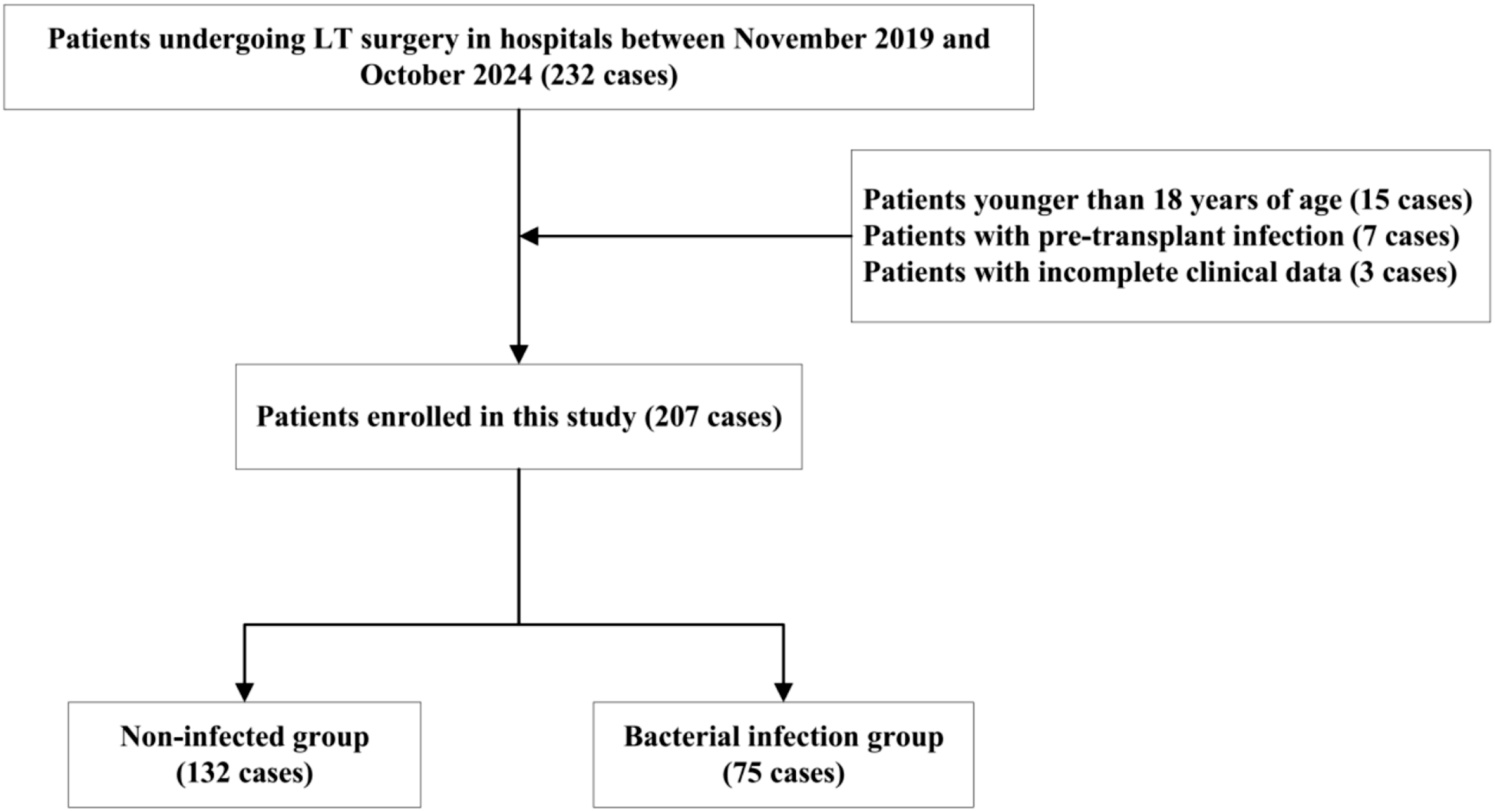
Flowchart of study subject selection process.

Based on the exclusion criteria, the following patients were excluded from the study: patients younger than 18 years of age (15 cases), those infected before transplantation (7 cases), and those with missing relevant clinical data or laboratory test results involved in this study (3 cases). Finally, 207 patients were enrolled in this study and categorized into the bacterial infection and non-infection groups according to the relevant criteria (Figure 1).

### 2.2 Definition and diagnostic criteria of infection

Early post-LT infections were defined as those occurring within the first month after LT. The diagnostic criteria for bacterial infection were as follows: (1) postoperative clinical symptoms such as chills, persistent fever (T > 38°C), coughing up sputum, shortness of breath, or sputum sounds; (2) positive results of bacterial cultures of blood, urine, sputum, or bronchoalveolar lavage fluid, bile, wound drainage fluid, or secretions; (3) imaging examinations (X-ray, ultrasound, CT, etc.) suggesting the presence of an infectious focus, which was characterized as the presence of chest CT or X-ray examination showing inflammatory infiltrative lesions in the lungs, with or without pleural effusion or abdominal ultrasound or CT showing fluid or abscess in the abdominal cavity.

### 2.3 Surgical and perioperative management

The LT was performed by the same transplant team, and all patients received allogeneic liver transplants by matching the donor and recipient blood groups. Carbapenem antibiotics were administered prophylactically 30 min before surgery, with an additional group administered if the length of surgery exceeded 3 h. Ertapenem was used on the first postoperative day to prevent bacterial infections and caspofungin to prevent fungal infections, and after 7 days of continuous use, the antibiotic grade was gradually reduced or combined with other types of antibiotics according to the patient’s condition and the results of specimen cultures, and cefoperazone sodium and sulbactam sodium was used routinely on an empirical basis. Postoperative drug regime included the routine use of tacrolimus, mycophenolate mofetil, adrenal glucocorticoid triple immunosuppressive therapy program, tacrolimus blood concentration of 8 ~ 12 μg/L in the first month after surgery, 6 ~ 8 μg/L in the second month, along with initial use of glucocorticoid (intravenous methylprednisolone). The dosage of glucocorticoids was gradually reduced, around the seventh day to change to oral prednisone tablets. The postoperative immunosuppressant dosage of the patients was adjusted individually according to the occurrence of infection and rejection. All patients received intensive care in the transplantation care unit after the surgery; prevention and control measures for catheter-related infections were strictly implemented and evaluated, and the catheters were removed as soon as possible.

### 2.4 Clinical data collection

General data included patients’ age, gender, body mass index (BMI), primary disease, diabetes mellitus, hypertension, hepatic encephalopathy, model for end-stage liver disease (MELD) score, child-Pugh score, donor’s age, donor’s gender, donor’s BMI, operative time, intraoperative blood transfusion volume, blood loss, cold ischemia time, ventilator use time, and postoperative ICU stay. We also investigated the pathogenic bacterial spectrum and infection distribution in patients with postoperative bacterial infections after LT.

Laboratory findings (last preoperative and postoperative day 1) consisted of white blood cell count (WBC), neutrophil count (N), lymphocyte count (L), C-reactive protein (CRP), procalcitonin (PCT), hemoglobin (HB), platelet count (PLT), albumin (ALB), alanine aminotransferase (ALT), total bilirubin (TB), serum creatinine (SCr), prothrombin time (PT), prognostic nutritional index (PNI), systemic immune inflammation index (SIl), neutrophil-to-lymphocyte ratio (NLR).

PNI, SII and NLR were calculated as follows: PNI = ALB + 5 × L; SII = PLT × N/L; NLR = N/L.

### 2.5 Establishment and evaluation of the prediction model

We used backward stepwise regression to determine the modeling variables, and R Studio 4.4.1 software to plot the nomogram. In backward stepwise regression, the optimal regression model is constructed by gradually removing the variables that contribute the least to the model, starting from the full model that contains all candidate variables. In each iteration, we set variables with *p*-values greater than 0.05 to be removed from the model until all remaining variables met the significance requirements. The performance of the nomogram was evaluated using the ROC curve and AUC to evaluate the discriminatory ability of the prediction model, and the AUC > 0.7 indicated that the model had a strong predictive value. Calibration curves were plotted based on predicted and true probabilities. The Hosmer–Lemeshow (H–L) test assessed the goodness of fit, and the H–L test combined with the calibration curves evaluated the calibration of the predictive model. Decision curve analysis (DCA) was performed to assess the clinical utility and validity of the model. K-fold cross-validation (dividing the data into k subsets, sequentially using each subset as the validation set and the remaining K-1 subsets as the training set, and calculating the average of the N validation results as the final performance metric) and bootstrap validation (from the original dataset with a put-back, random sampling to generate multiple new training sets, random sampling, generate multiple new training sets, the unsampled samples form the validation set, repeat N times, and compute the average performance) for model validation. We set K to 10, and the total number of iterations was 207 and 1,000, respectively, to compute the average AUC value.

### 2.6 Statistical analysis

SPSS 29.0 software was used for statistical analysis. Measures that conform to a normal distribution are denoted by mean and standard deviation, and an independent sample *t*-test was used for comparison between groups. The measurement data of non-normal distribution were expressed as median and quartile (the first quartile and the third quartile), and a non-parametric rank sum test was used for comparison between groups. The count data were expressed as rates or percentages, and the chi-square test was used for comparison between groups. Univariate analysis was used to screen the influencing factors of bacterial infection. Binary logistic regression was used to analyze the independent risk factors of bacterial infection after LT. The Youden index was used to determine the optimal critical value of independent risk factors. *P* < 0.05 was considered statistically significant.

## 3 Results

### 3.1 Clinical characteristics of patients

Among the 207 LT patients included in this study, 161 (77.8%) were male and 46 (22.2%) were female, with a mean age of 48.96 ± 9.47 y. The primary disease was cirrhosis in 122 cases (58.9%), including hepatitis B cirrhosis in 60 cases (29%), primary biliary cirrhosis in 14 cases (6.8%), alcoholic cirrhosis in 14 cases (6.8%), autoimmune hepatitis cirrhosis in 14 cases (6.8%), mixed cirrhosis in 9 cases (4.3%), occult cirrhosis in 6 cases (2.9%), hepatitis C cirrhosis in 4 cases (1.9%), portal cirrhosis in 1 case (0.5%), primary disease as hepatocellular carcinoma in 62 cases (30%), liver failure in 12 cases (5.8%), hepatolenticular degeneration in 3 cases (1.4%), hepatic hemangioma in 2 cases (1%), hilar cholangiocarcinoma in 2 cases (1%), calculus of intrahepatic duct in 2 cases (1%), polycystic liver disease in 1 case (0.5%), and Budd-Chiari syndrome in 1 case (0.5%).

### 3.2 Distribution characteristics of pathogenic bacteria in bacterial infection

**Figure 2:**
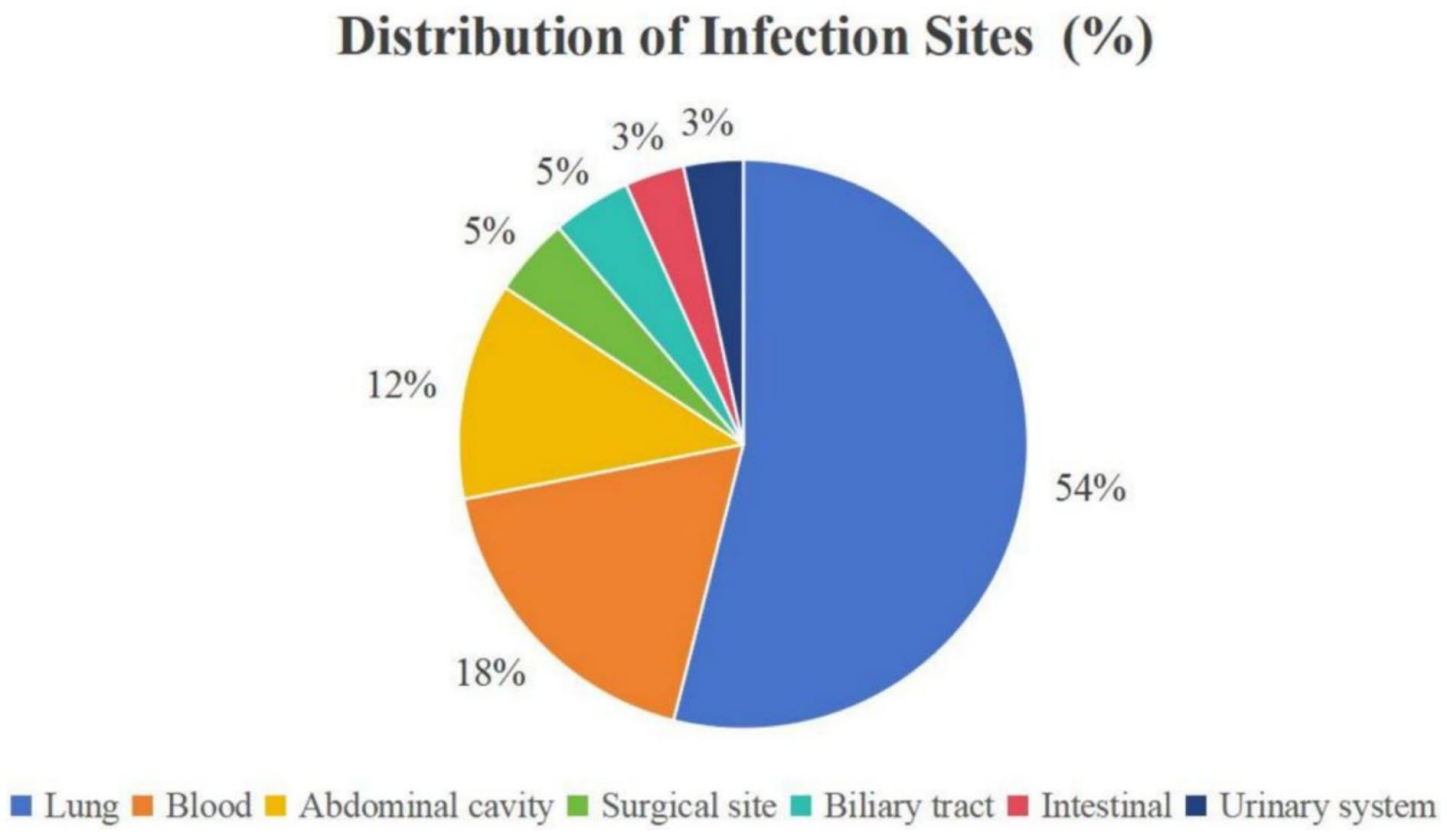
Distribution of infection sites.

Among the 207 LT patients, 75 had early postoperative bacterial infections, with an incidence of 36.2%. A total of 89 strains of non-repetitive pathogens were detected in 75 patients, including 57 strains of Gram-negative bacteria (64.04%), 17 strains of *Acinetobacter baumannii* (19.10%), 10 strains of *Escherichia coli* (11.24%), six strains of *Stenotrophomonas maltophilia* (6.74%), five strains of *Pseudomonas aeruginosa* (5.62%), and five strains of *Klebsiella pneumoniae* (5.62%). There were 32 strains of Gram-positive bacteria (35.96%), including nine strains of *Staphylococcus epidermidis* (10.11%), eight strains of *Enterococcus faecium* (8.99%), and six strains of *Staphylococcus aureus* (6.74%) (Table 1). The infection sites were mainly concentrated in the lungs (53.93%), blood (17.98%), and abdominal cavity (12.36%) (Figure 2).

**Table 1:** Distribution of pathogenic bacteria of bacterial infection after LT.

Pathogenic bacteria	Number of plants	Composition ratio (%)
Gram-negative bacteria	57	64.04
*Acinetobacter baumannii*	17	19.10
*Escherichia coli*	10	11.24
*Stenotrophomonas maltophilia*	6	6.74
*Klebsiella pneumoniae*	5	5.62
*Pseudomonas aeruginosa*	5	5.62
Others	14	15.7
Gram-positive bacteria	32	35.96
*Staphylococcus epidermidis*	9	10.11
*Enterococcus faecium*	8	8.99
*Staphylococcus aureus*	6	6.74
Others	9	10.1
Total	89	100

### 3.3 Univariate analysis of general baseline data and preoperative variables

#### 3.3.1 Comparison of preoperative data

The results of univariate analysis of preoperative data showed that the differences in preoperative MELD score ≥ 25, and the preoperative NLR, SII and ALB levels were statistically significant in the infected group compared with the uninfected group (*p* < 0.05). The differences in patients’ sex, age, BMI, history of hypertension, history of diabetes mellitus, hepatic encephalopathy, ascites, Child-Pugh score, WBC count, and HB, PLT, ALT, TB, SCr, PT, CRP, PCT, and PNI levels were not statistically significant (*p* > 0.05) (Table 2).

**Table 2:** Univariate analysis of general baseline information and preoperative variables.

Variables	Non-infected group (132 cases)	Bacterial infection group (75 cases)	*p*-value
Gender (%)			0.817
Male	102 (77.27%)	59 (78.67%)	
Female	30 (22.73%)	16 (21.33%)	
Age (years)	49.3 ± 9.68	48.37 ± 9.06	0.376
BMI (kg/m^2^)	22.94 (20.67, 25.95)	23.29 (20.88, 25.56)	0.781
Hypertension (%)	11 (8.33%)	9 (12%)	0.391
Diabetes (%)	16 (12.12%)	14 (18.67%)	0.198
Hepatic encephalopathy (%)	7 (5.30%)	2 (2.67%)	0.590
Ascites (%)	99 (75%)	56 (74.67%)	0.958
MELD score (points)			0.017*
<25	117 (88.64%)	57 (76%)	
≥25	15 (11.36%)	18 (24%)	
Child-Pugh score			0.126
A	38 (28.79%)	19 (25.33%)	
B	57 (43.18%)	25 (33.33%)	
C	37 (28.03%)	31 (41.33%)	
WBC (×10^9^/L)	4.09 ± 2.56	4.61 ± 3.20	0.204
ALB (g/L)	36.21 ± 6.75	33.88 ± 6.47	0.016*
CRP (mg/L)	3.99 (1.26, 10.18)	5.9 (1.16, 17.05)	0.121
PCT (ng/mL)	0.10 (0.06, 0.24)	0.12 (0.06, 0.35)	0.290
NLR	2.86 (2.4, 33)	4.52 (2.8, 6.89)	<0.001*
PNI	40.04 ± 7.58	38.12 ± 7.35	0.077
SII	186.38 (104.74, 287.4)	304.79 (174.25, 627.62)	<0.001*
HB (g/L)	109 (90.75, 133.5)	101 (86, 128)	0.225
ALT (U/L)	32 (19.5, 50.75)	33 (22, 57)	0.806
TB (μmol/L)	38.1 (22.43, 71.55)	45.6 (25.6, 112.6)	0.182
SCr (μmol/L)	66.5 (57, 84)	67 (54, 90)	0.970
PT(s)	13.75 (11.8, 16.38)	14.9 (11.8, 18.30)	0.050
PLT (×10^9^/L)	62 (44.5, 91.75)	66 (41, 111)	0.103

*p < 0.05 was considered statistically significant.

WBC, white blood cell count; CRP, C-reactive protein; PCT, procalcitonin; HB, hemoglobin; PLT, platelet count; ALB, albumin; ALT, alanine aminotransferase; TB, total bilirubin; SCr, serum creatinine; PT, prothrombin time; PNI, prognostic nutritional index; SII, systemic immune inflammation index; NLR, neutrophil-to-lymphocyte ratio.

#### 3.3.2 Comparison of intraoperative data

A comparison of intraoperative variables showed that the bacterial infection group had a higher operative time, intraoperative blood transfusion and blood loss than the uninfected group, and the difference was statistically significant (*p* < 0.05). The differences in donor age, sex, body mass index, and cold ischemia time were not statistically significant (*p* > 0.05) (Table 3).

**Table 3:** Univariate analysis of intraoperative variables.

Variables	Non-infected group (132 cases)	Bacterial infection group (75 cases)	*P*-value
Age of donor (years)	45.39 ± 14.29	43.53 ± 12.93	0.373
Donor gender (%)			0.229
Male	114 (86.36%)	60 (80%)	
Female	18 (13.64%)	15 (20%)	
Donor BMI (kg/m^2^)	23.01 ± 3.58	26.00 ± 23.76	0.063
Cold ischemia time (minutes)	183.79 ± 52.76	197.37 ± 64.42	0.384
Operation time (minutes)	390 (350, 430)	447.50 (390, 541.25)	0.001*
Intraoperative blood transfusion volume (mL)	2,200 (1,200, 3,800)	3,850 (2,400, 5562.50)	0.001*
Intraoperative blood loss (mL)	1800 (1,000, 2,700)	2,950 (1,500, 5625.00)	0.001*

*p < 0.05 was considered statistically significant.

#### 3.3.3 Comparison of postoperative data

Comparison of postoperative variables showed that, compared with the uninfected group, the bacterial infection group showed statistically significant differences in the postoperative ICU stay, postoperative ventilation time, biliary complications, CRRT, reoperation, ALB, HB, ALT, SCr, PT, NLR, and PNI levels (*p* < 0.05). The differences in CRP, PCT, SII, WBC, PLT, and TB levels were not statistically significant (*p* > 0.05). Postoperative PNI and ALB levels were lower than preoperative levels, while CRP, PCT, NLR, SII, and WBC counts were higher than preoperative levels (Table 4).

**Table 4:** Univariate analysis of postoperative variables.

Variables	Non-infected group (132 cases)	Bacterial infection group (75 cases)	*P*-value
Length of ICU stay (days)	9 (7, 11)	10 (7, 18)	0.005*
Ventilator time (hours)	17 (0, 22)	21 (11, 96)	0.001*
Biliary complications (%)	8 (6.06%)	11 (14.67%)	0.039*
CRRT (%)	10 (7.58%)	13 (17.33%)	0.032*
Re-operation (%)	3 (2.27%)	15 (20%)	<0.001*
WBC (× 10^9^/L)	7.56 (5.18, 10.54)	7.93 (4.43, 10.33)	0.898
ALB (g/L)	36.03 ± 3.92	33.64 ± 6.02	0.001*
CRP (mg/L)	51.54 (32.53, 73.42)	41 (22.41, 66.26)	0.056
PCT (ng/mL)	5.22 (2.21, 15.72)	12.97 (4.03, 29.57)	0.065
NLR	26.60 (18.64, 32.06)	31.22 (18.52, 48.11)	0.026*
PNI	37.85 (34.35, 40.49)	35.05 (31.85, 39.05)	<0.001*
SII	1563.05 (902.9, 2263.2)	1561.11 (789.6, 3830.59)	0.318
HB (g/L)	101.5 (87.25, 109)	91 (77, 106)	0.006*
ALT (U/L)	294.5 (165, 453)	410 (181, 820.50)	0.010*
TB (μmol/L)	46.85 (30.08, 86.58)	53.10 (33.25, 94.10)	0.394
SCr (μmol/L)	86 (70, 113.25)	110 (80.50, 145.50)	0.002*
PT(s)	13.55 (12.70, 15.20)	14.80 (13.40, 17.60)	<0.001*
PLT (×10^9^/L)	55 (36.25, 88.5)	50 (31.50, 80)	0.233

*P < 0.05 was considered statistically significant.

WBC, white blood cell count; CRP, C-reactive protein; PCT, procalcitonin; HB, hemoglobin, PLT, platelet count; ALB, albumin; ALT, alanine aminotransferase; TB, total bilirubin; SCr, serum creatinine; PT, prothrombin time; PNI, prognostic nutritional index; SII, systemic immune inflammation index; NLR, neutrophil-to-lymphocyte ratio.

### 3.4 Multivariate analysis of bacterial infection after liver transplantation

Binary logistic regression analysis showed that operative time (*p* = 0.005), ventilator use time (*p* = 0.025), length of postoperative ICU stay (*p* = 0.015), preoperative SII (*p* = 0.001), and postoperative NLR (*p* = 0.024) were independent risk factors for the development of bacterial infections in the early postoperative period after liver transplantation (Table 5). Using the maximum vertical distance between the ROC curve and the diagonal line, the thresholds for the above risk factors for bacterial infections were determined to be operative time greater than 7 h, ventilator use greater than 58 h, postoperative ICU stay greater than 14 days, SII greater than 270, and NLR greater than 33 (*p* < 0.05).

**Table 5:** Logistic multivariate analysis of related factors affecting early postoperative bacterial infection.

Variable	*β*	SE	Wald	*P*	OR	95%CI
MELD score (points)	−0.518	0.549	0.887	0.346	0.596	(0.203, 1.749)
SII	0.003	0.001	11.778	0.001*	1.003	(1.001, 1.005)
Operation time (minutes)	0.008	0.003	7.926	0.005*	1.008	(1.002, 1.014)
Intraoperative blood loss (mL)	0.483	0.472	1.048	0.306	1.621	(0.643, 4.088)
Length of ICU stay (days)	0.118	0.049	5.893	0.015*	1.125	(1.023, 1.237)
Ventilator time (hours)	0.013	0.006	5.024	0.025*	1.013	(1.002, 1.024)
NLR	0.017	0.007	5.097	0.024*	1.017	(1.002, 1.031)
PNI	−0.063	0.047	1.784	0.182	0.939	(0.857, 1.030)

*P < 0.05 was considered statistically significant.

### 3.5 Development and validation of the prediction model

**Figure 3:**
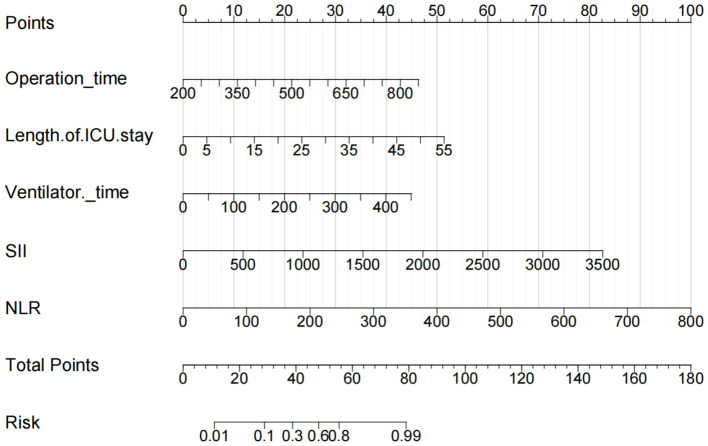
Nomogram of bacterial infection after LT. The points from each of the four components of the nomogram: Operation time (50 min = 4 points), Length of ICU stay (5 days = 5 points), ventilator time (50 h = 5 points), NLR (0 = 0 points, 100 = 12.5 points), SII (0 = 0 points, 500 = 12 points).

Based on the results of multifactorial analysis, the R Studio software was used to construct and build a nomogram prediction model for early postoperative bacterial infection in liver transplantation patients (Figure 3), and the results of the nomogram model showed that: for every 50 min increase in the operation time, the nomogram model increased by 4 points, for every 5 days increase in the postoperative ICU hospitalization, the nomogram model increased by 5 points, and for every 50 h increase in the postoperative mechanical ventilation time, the nomogram model increased by 5 points, for every 100 increase in NLR, the nomogram model increased by 12.5 points, and for every 500 increase in SII, the nomogram model increased by 12 points. By substituting the values of each influencing factor into the model and analyzing and accumulating the corresponding individual scores, the final calculated total score corresponds to the risk value at the bottom, which is the risk probability of early postoperative bacterial infection in LT patients.

**Figure 4:**
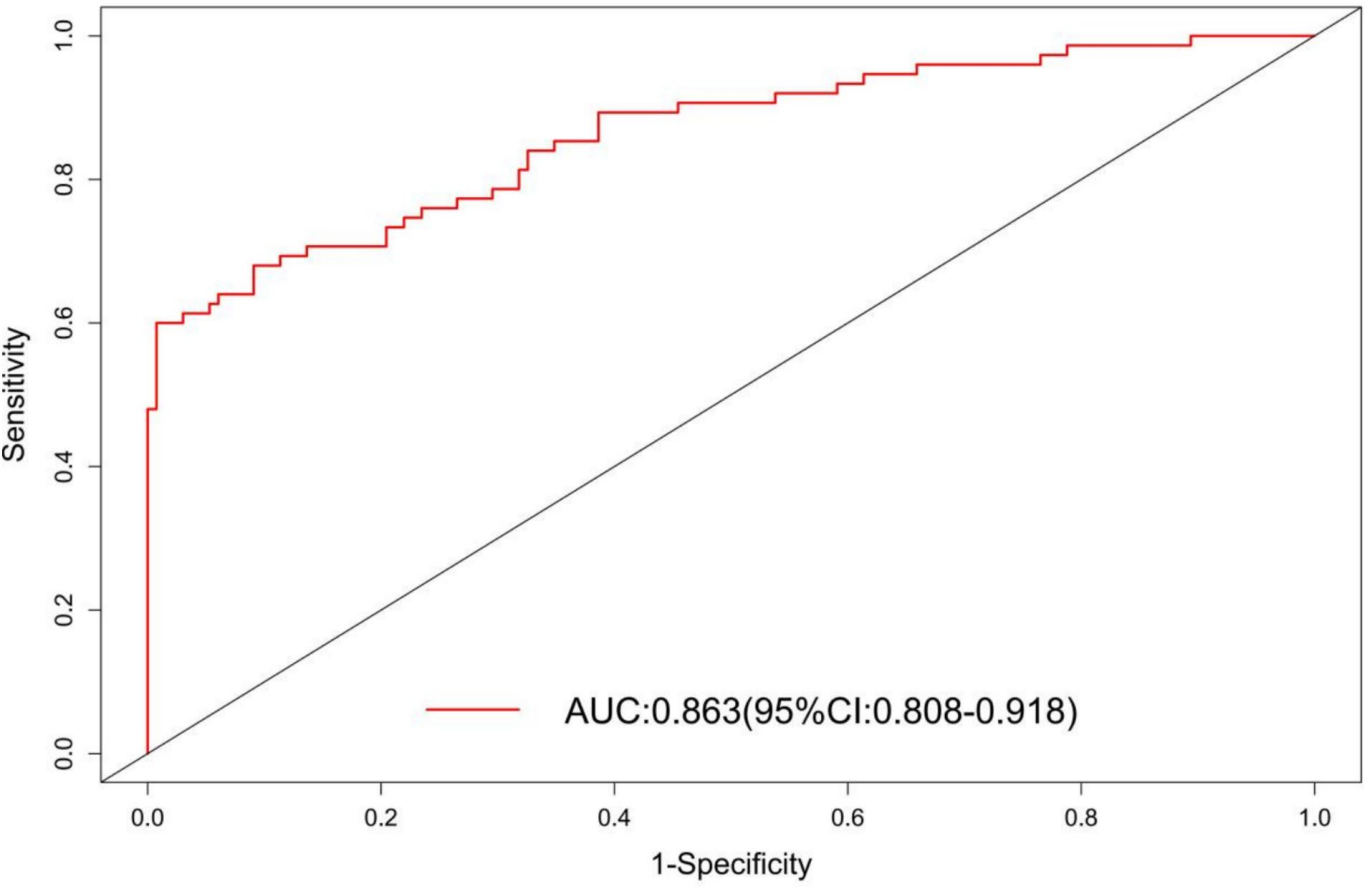
ROC curve of bacterial infection prediction model after LT.

**Figure 5:**
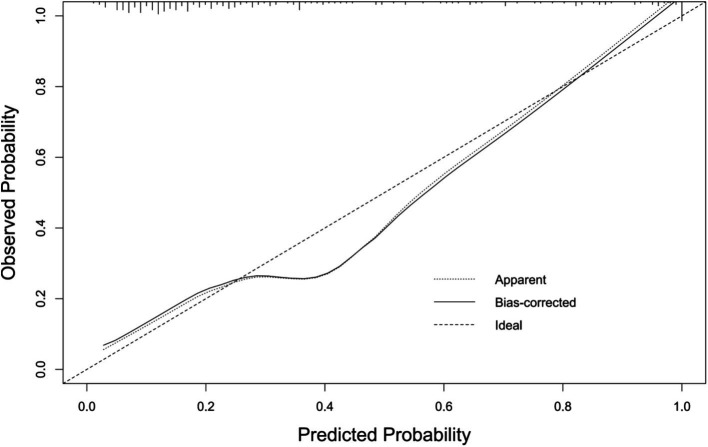
Calibration curve of bacterial infection prediction model after LT.

**Figure 6:**
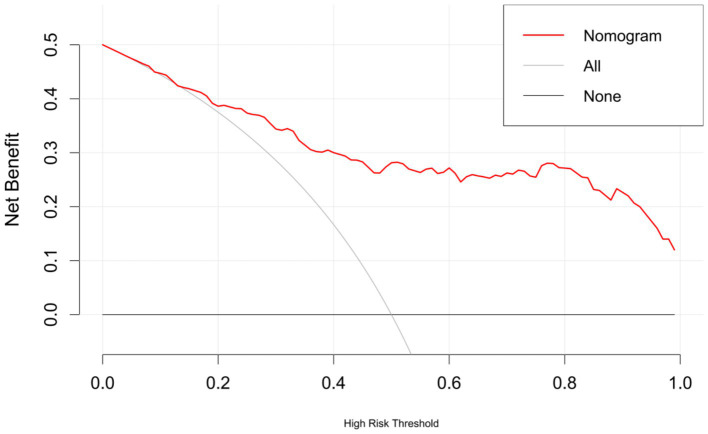
Decision curve analysis (DCA) curve of bacterial infection prediction model after LT.

The AUC of the nomogram was 0.863 (95%CI: 0.809–0.918), indicating good discriminatory ability of the model (Figure 4). The H–L test showed that the predicted values of the model fit well with the actual data (*χ*^2^ = 6.636, *p* = 0.576), and the calibration curve showed a mean error of 0.044, indicating good agreement between the predicted probabilities and the actual probabilities (Figure 5). The decision curve of the nomogram lies above the two extreme curves within a threshold range of >14% (Figure 6), indicating good clinical benefit in this range. We used 10-Fold cross validation and Bootstrap validation for model validation, where the number of failed iterations was zero. The calculated AUCs were 0.842 and 0.854, respectively, indicating that the developed model was reproducible, had good resolution, and had a high degree of compliance.

## 4 Discussion

Bacterial infection is a common complication in the early postoperative period after LT. In this study, the rate of postoperative bacterial infection in LT patients was 36.2%, which is similar to that reported in previous studies. However, a multicenter, large-sample study conducted in Spain showed a 41.3% incidence of postoperative bacterial infections after LT. This discrepancy may be related to regional differences and alcoholic liver disease as the main etiology (18). In India, the incidence of postoperative bacterial infections after LT was 42.9–75%, which is not only due to the different sources of liver supply but also considered to be related to the slightly older age of the study and the conditions and level of medical management of patients in the perioperative period of LT (19). This indicates that there may be differences in the incidence of postoperative bacterial infections after LT among countries, sample sizes, patient sources, main etiologic factors, and follow-up times.

Various reasons, such as the antibiotic regimen of each transplant center, whether or not gut decontamination is performed preoperatively, and the distribution of common pathogens of hospital-acquired infections, can lead to differences in the major sites and pathogens of postoperative infections in LT; however, Gram-negative versus Gram-positive organisms are still the major causative agents. A Korean study found that Gram-negative bacteria were more common than Gram-positive bacteria in infections (20). This is consistent with the results of the present study, where Gram-negative bacteria (64.04%) accounted for more than Gram-positive bacteria (35.96%), whereas more than 10 years ago, post-transplantation infections were predominantly characterized by Gram-positive pathogens (21), which may be attributed to the fact that with the widespread use of antibiotics and newer iterations of antibiotics, the spectrum of pathogens has been constantly changing. Our pathogenic bacterial culture results showed that *Acinetobacter baumannii* was the most common pathogen for postoperative bacterial infections in LT (19.10%), whereas drug sensitivity tests revealed that some of the bacteria were either highly resistant or multi-drug resistant. In a 2018 ~ 2022 study of hospital-acquired infections in LT patients (22), *Klebsiella pneumoniae* and *Acinetobacter baumannii* were the main causative organisms and showed high resistance to carbapenem antibiotics. Another study showed that the cumulative mortality rates of patients with carbapenem-resistant *Acinetobacter baumannii* infections after LT were 58.6, 65.5, and 65.5% on postoperative days 5, 10, and 30, respectively (23). In this situation, active pathogenetic surveillance of LT patients to understand the trends in pathogen distribution and drug resistance is essential for the rational use of antimicrobial drugs and the timely adoption of preventive and control measures against drug-resistant bacteria.

The pulmonary infection (53.93%) was the main site of infection, followed by the blood and abdominal cavities. This is generally in agreement with the results of previous reports (24–26). Factors such as prolonged ventilator-assisted ventilation, multiple tracheal intubations, and a history of smoking may cause damage to patients’ respiratory mucosa, weakening the barrier function, and sputum expulsion, which in turn leads to susceptibility to respiratory infections in the postoperative period of LT. In addition, *Acinetobacter baumannii* can utilize short fimbria-like projections on the surface of bacterial cells to adsorb to bronchial epithelial cells, resulting in the lungs being a common focus for *Acinetobacter baumannii* infections (27).

The technical difficulty and complexity of transplantation surgery and prolonged operative time lead to an increase in the duration of continuous opening of the abdominal cavity, which is the main cause of surgical site infection after transplantation (28). Wu et al. (29) concluded that the prolongation of the LT operative time implies severe trauma and intestinal bacterial translocation, which significantly increases the rate of patients’ postoperative Gram-negative infections, and further studies showed that an operation time ≥ 8 h was a high-risk factor for secondary bacterial infections in the abdominal cavity after LT (30). Liu et al. (31) showed that surgical time > 400 min was an independent influencing factor for postoperative bacterial infections after LT, which was in agreement with the results of our study, which showed that surgical time was significantly increased in the infected group compared to that in the non-infected group, and that the length of liver transplantation surgery was more than 7 h as a threshold for predicting postoperative bacterial infections. In addition to factors such as the skill level of the surgeon and the degree of teamwork, the duration of surgery is also associated with the degree of intraoperative bleeding. Most patients develop coagulation disorders and significant intraoperative blood loss. In our study, the average operative time for patients with blood loss of >10 L was approximately 11 h. In the postoperative period, 89% of patients developed bacterial infections. A cohort study found that for every 1-min increase in operative time, the probability of prolonging the patient’s ICU stay increased by 0.4% (32), which also indirectly increased the risk of infection. Therefore, preoperative coagulation evaluation of LT patients and a multidisciplinary discussion of the surgical plan and operation details are the best ways to shorten the operative time and reduce the incidence of postoperative bacterial infections, which have a positive impact on improving the prognosis of transplant patients.

In patients with end-stage liver disease, malnutrition and sarcopenia often occur simultaneously because of the chronic decompensated state of the disease, resulting in a continuous decline in activity and varying degrees of muscle atrophy and fatigue. When the patient’s respiratory muscles are atrophied and weak, it is very easy for the patient to become ventilator-dependent, making it difficult to pass the spontaneous breathing test (SBT), which prolongs the duration of mechanical ventilation and leads to ventilator-associated lung injury and pneumonitis. Previous studies (33, 34) have shown that postoperative mechanical ventilation for more than 24 h causes damage to the airway mucosal barrier, increases the risk of early postoperative lung infections, and is one of the main causes of early postoperative sepsis and death in LT patients. In our study, ventilator use for >58 h was critical for postoperative bacterial infection in LT patients. The removal of tracheal intubation within 8 h after transplantation is an effective measure to reduce the incidence of postoperative bacterial infections in the lungs (35). Therefore, early tube removal can reduce ventilator dependence while ensuring that the patient’s respiratory function is tolerable, and the implementation of respiratory exercises can facilitate pulmonary rehabilitation.

Patients with early post-transplant bacteremia have an approximately two-fold increase in the length of hospitalization and ICU stay and a higher mortality rate than uninfected patients (36). Postoperative infections can exacerbate the condition, making treatment more difficult, and prolonging the course of the disease. Bacterial infection is an independent predictor of prolonged hospitalization in LT patients (37). We found that the incidence of bacterial infections was higher in patients with a postoperative ICU stay >14 days; however, in a study by Li et al. (38) scholars, an ICU stay >9 days was an independent risk factor for postoperative bacterial infections. The difference in findings may be related to the increase in ICU length of stay due to different patient management protocols at transplant centers. Active optimization of the perioperative management process by the transplant team to improve the quality and speed of recovery is an effective measure for shortening the ICU and total hospital stay of LT patients.

Conventional inflammatory cells reflect the immune status of the body to a certain extent; however, the reintegrated and calculated inflammatory indices can reflect the immune status of the body more comprehensively than the individual indices. NLR and SII are inflammatory markers based on the calculation of peripheral blood neutrophil count, lymphocyte count, and PLT (39, 40); they are simple to calculate, easy to test, inexpensive, and have been used as prognostic indices in a variety of diseases (41–43). Normally, neutrophil count increases as inflammatory diseases worsen. However, in cases of severe infection or extreme wasting, the neutrophil count may not increase, leading to “false-negative” results when assessing the disease progression. The lymphocyte count, on the other hand, reflects the patient’s immune status and usually decreases as the inflammatory disease worsens. However, this decrease is gradual and therefore does not rapidly reveal the extent of changes in the body’s infections. The NLR, a composite indicator of inflammation in the body, is superior to neutrophils and lymphocytes alone in predicting disease progression (44). Compared to conventional infection markers such as IL-6, CRP, and WBC, NLR exhibits a high degree of sensitivity, specificity, and positive rate for bacteremia (45) and is not affected by the type of transplanted organ or the patient’s sex (46). Park et al. (36) demonstrated that the preoperative NLR was positively correlated with the incidence of bacteremia and mortality in the early postoperative period and was an independent predictor of early postoperative infection. In this study, WBC, PCT, and CRP showed no significant differences between the two groups, whereas preoperative and first-day postoperative NLR were strongly associated with early postoperative bacterial infections after LT Further multifactorial analysis showed that NLR on the first postoperative day was an independent high-risk factor for early postoperative bacterial infections after LT. This is consistent with the findings of Tu et al. (47) that NLR on the first postoperative day after LT can reflect the risk of infection development in patients earlier than the positive results of graft preservation fluid culture.

In the early stage of inflammation, neutrophils phagocytose pathogens (48), and activated PLT interact with monocytes and lymphocytes to exacerbate the inflammatory response (49); therefore, an elevated SII indicates that the patient’s immune and inflammatory systems are in a state of imbalance and that the intensity of the inflammatory response is higher than the immune function. In a Korean study, the incidence of post-transplant sepsis in patients with preoperative SII ≥870 was 59.1%, with a high mortality rate of 76.9% (50), suggesting that patients with higher SII are more likely to be intolerant of infections, and are a possible cause of the high mortality rate. Other investigators (51) utilized the SII in combination with other test results to evaluate postoperative abdominal infections after LT, and the results suggested a good predictive value for postoperative abdominal infections. Our results were in general agreement with the results of the above studies that preoperative SII ≥270 was an independent risk factor for early postoperative bacterial infection after LT. In patients with LT, assessment of preoperative SII levels may provide a reference for clinical screening of high-risk groups for postoperative bacterial infection.

In our study, CRP and PCT did not show predictive value as traditional inflammatory markers, which may be related to the fact that high doses of hormones and immunosuppressant use after transplantation affect the status of the transplanted liver and CRP synthesis, which was reduced by immunosuppressants that block reactive T signaling (52). PCT in non-infectious systemic inflammatory response syndromes such as surgery trauma, non-specifically elevated; thus, there were no significant differences between group comparisons. In conclusion, NLR and SII, as comprehensive indicators of the inflammatory and immune status of patients, can indicate the prognosis of patients and the choice of antibiotic management regimen earlier than the results of pathogenic bacterial cultures by monitoring the levels of NLR and SII in patients (40).

Considering the specificity of the immune status of transplant patients and the increased incidence of infections with multidrug-resistant bacteria, the development of an effective predictive model for postoperative bacterial infections during LT is of great clinical importance. A nomogram enables clinicians to predict the likelihood of specific clinical events based on individual variables. In this study, we identified the preoperative SII, duration of surgery, ventilator use time, length of postoperative ICU stay, and NLR on the first postoperative day as independent risk factors for postoperative bacterial infections after LT We constructed a prediction model based on the five factors that had an AUC of 0.863, demonstrating excellent performance in discriminating between patients who would or would not develop bacterial infections after LT. According to the risk parameters in the model, individualized prediction and assessment of the risk of developing bacterial infection after liver transplantation can provide a reference basis for antibiotic selection and immunomodulation.

This study has certain limitations. First, this was a single-center study that may have differed from other centers in terms of the surgical technique, pathogen distribution, and postoperative therapeutic care regimens. Second, the study data were collected retrospectively from patients’ medical records, which may not contain all the necessary information for analysis and may be subject to selection bias. Finally, owing to the small sample size, we only performed internal validation, and the generalization of our findings may be limited; external validation and multicenter collaborations will be needed to validate our findings in the future.

## 5 Conclusion

The risk-warning model constructed in this study integrates preoperative SII and postoperative NLR for the first time. The model is simple and easy to operate and the prediction results are intuitive and readable, which can help medical staff recognize high-risk patients at an early stage. In addition, to reduce the rate of early post-transplant bacterial infection, the transplantation team should make adequate preoperative preparations to keep the operative time within 7 h as much as possible. Furthermore, if the patient’s postoperative respiratory condition is good, the ventilator should be removed within 48 h, and efforts should be made to shorten the patient’s hospitalization in the ICU, while focusing on the evaluation of the patients with high levels of SII and NLR.

## Data Availability

Publicly available datasets were analyzed in this study. This data can be found: the original contributions presented in the study are included in the supplementary material, further inquiries can be directed to the corresponding author.
